# Supplementary material for: Neural dynamics of phoneme sequences reveal position-invariant code for content and order
Source: Nat Commun. 2022 Nov 3;13:6606. doi: 10.1038/s41467-022-34326-1 (PMC9633780; doi:10.1038/s41467-022-34326-1)
Supplement: Supplementary file 1 — Supplementary Information [file 41467_2022_34326_MOESM1_ESM.pdf]

1. Supplementary Notes

In addition to the analyses presented in the main text, we conducted a series of analyses presented here. They can be organised into four types: (i) comparisons between different types of decoding algorithms to validate our approach; (ii) manipulation of signal to noise ratio through the addition or noise or reduction of trial count to ensure that main claims remain true; (iii) additional analyses of interest, which probe representations and representational dynamics; (iv) exploration of the spatial evolution of representations through space, by analysing model beta coefficients.

1.1. Validation of analysis approach

1.1.1. Back-to-back regression

Comparing logistic decoding of phonetic features to the back-to-back regression algorithm.

1.2. SNR manipulation

1.2.1. Signal to noise ratio manipulation on acoustic generalisation analysis

Testing whether SNR affects decoding dynamics from acoustic signal.

1.2.2. Strength of MEG signal and its relation to decoding performance

Testing whether SNR affects decoding dynamics from neural signal.

1.2.3. Analysis on equalised trial counts

Replicating decoding analysis on equalised trial counts across phoneme positions.

1.3. Additional analyses of interest

1.3.1. Sequence analysis on mel spectrogram

Applying decoding analysis to the auditory signal.

1.3.2. History and future decoding

Testing hypotheses of sequence representations.

1.3.3. Sequence representation for different phonetic features

Confirming that decoding dynamics replicate across feature types.

1.3.4. Dynamics at sentence onset and sentence offset

Testing the dynamics of processing depending on global predictability.

1.3.5. Testing granularity of representation

Comparing representational formats that correlate with phonetic features.

1.4. Spatial evolution of encoding

1.4.1. Beta coefficients of decoding model

Timecourse of model coefficients over space, for each feature in the model.

Validation of analysis approach

1.1.1 Validation of back-to-back regression

Here we compare the results of the more classic logistic regression analysis with the results of the back-to-back regression we employ in this paper. The results are comparable, with the advantage of B2B providing cleaner, stronger estimates with less variance.

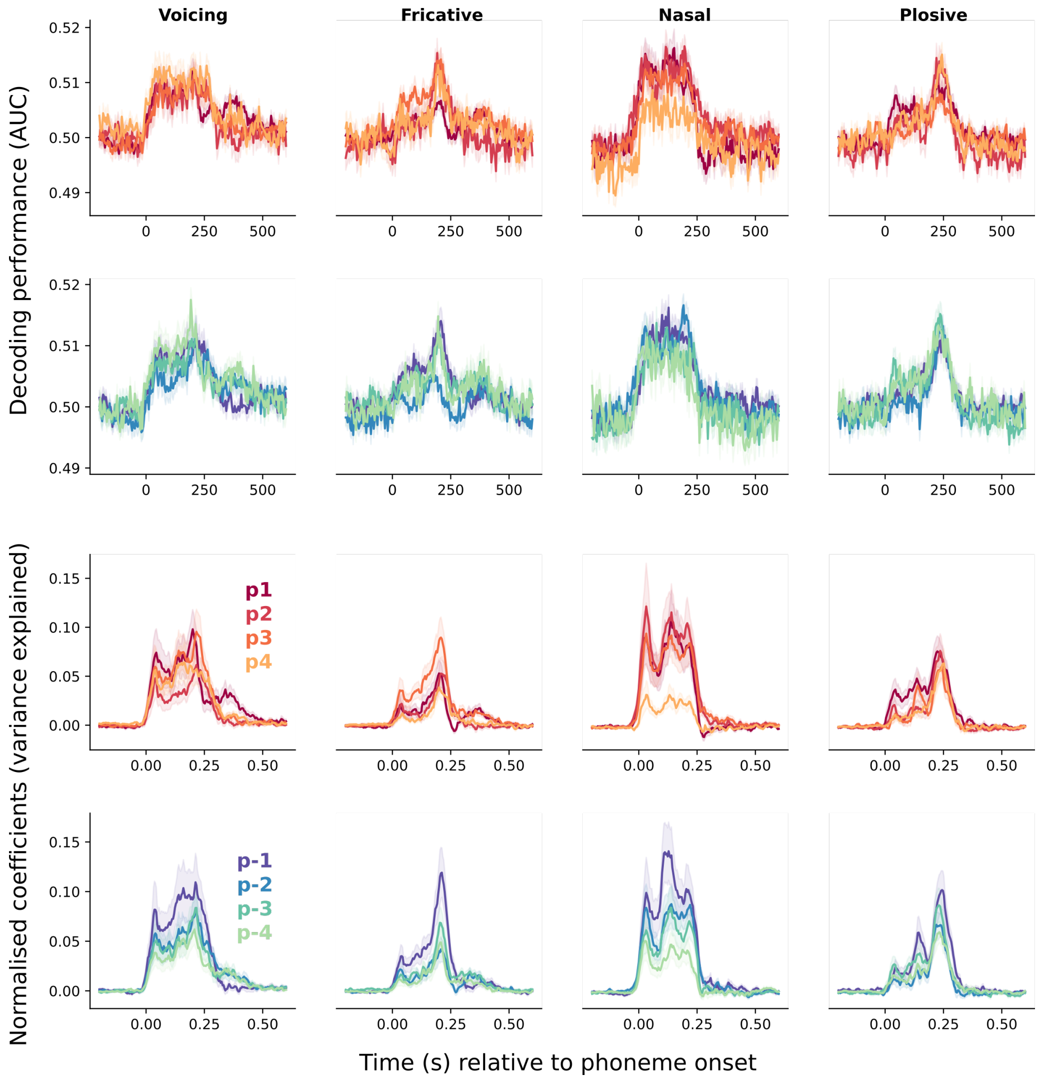

Supplementary Figure 1: **Comparing logistic regression to back-to-back regression.** Above: Results of decoding four phonetic features using 'classic' logistic regression decoding analysis with AUC as the performance metric. Each line corresponds to a different phoneme location in the word. Below: Same analysis when using back-to-back regression. Shading in the two plots corresponds to the standard error of the mean across 21 participants.

SNR manipulation

1.2.1. Signal to noise ratio manipulation on acoustic generalisation analysis

To test whether the sustained generalisation we observe in the auditory analysis is due to the relatively high SNR as compared to the MEG data, we re-ran the generalisation analysis when adding different levels of noise to the mel spectrogram. We observe a sustained decoding pattern at all SNR levels.

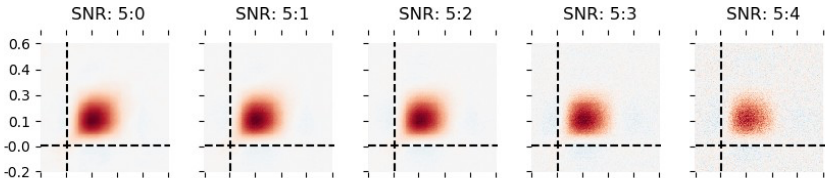

Supplementary Figure 2: **SNR manipulation on auditory mel spectrogram decoding.** x-axis corresponds to time in seconds relative to training the classifier. The y-axis corresponds to time in seconds relative to testing the classifier.

1.2.2. Strength of MEG signal and its relation to decoding performance

One potential confound in decoding performance is the overall signal strength (e.g. magnitude of the MEG response). It is possible that stimulus features which lead to larger responses will, in turn, aid better decodability of the other features encoded in that response. To test whether this was the case, we computed the root mean square (RMS) of the sensor data and attempted to decode this from the z-scored MEG responses, from 200 ms to 1000 after to each phoneme onset. For this, we used a Ridge regression decoder with Spearman R as the performance metric. First, we found that overall signal strength did not show the temporal dynamics that were elicited by any of our stimulus features of interest (see below figure). Second, we fit a mixed effects regression model between the single-trial RMS and decoding accuracy, for phonation, manner, and place of articulation. We modelled random slopes per participant and repetition. There was no significant relationship for any of the three features p’s > .3. Overall this suggests that the features we show to interact with phonetic encoding strength (e.g. surprisal and entropy) cannot be explained by the global strength of the signal. We believe the lack of effect may be due to our stimuli: continuous speech does not elicit clear evoked responses, and so single-trial variability in signal strength is negligible.

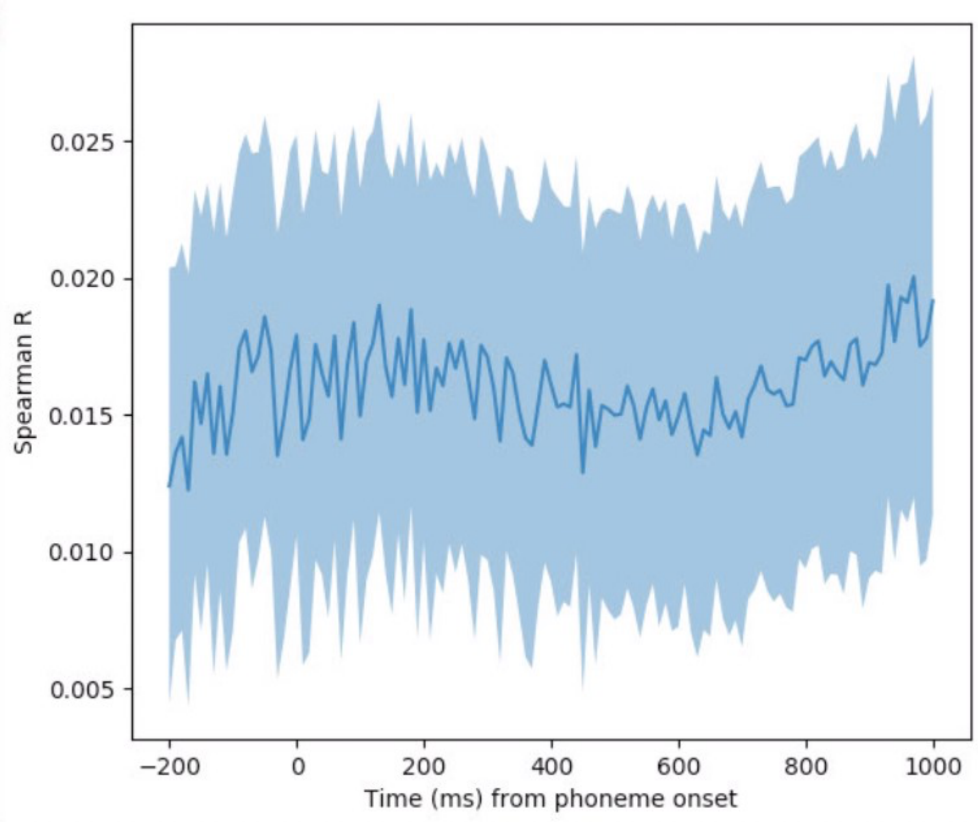

Supplementary Figure 3: **Decoding MEG signal strength.** Center trace represents average decoding performance over 21 participants. Shading represents standard error of the mean over participants.

1.2.3. Analysis on equalised trial counts

Due to the natural variation in word length, there were different numbers of trials included in the decoding analysis at phoneme positions. Maximum numbers of trials at the word boundaries (because all words have an onset and an offset) and gradually decreasing numbers of trials as the position moves further from the boundary. Estimates of decodability are noisier for phoneme positions with fewer trials. To allow direct comparisons of decoding strength across positions, we re-ran the analysis matching the number of trials to the fourth phoneme position (about 1500 trials).

Once the number of trials was equalised across positions, there were no significant differences in the strength of phoneme decoding. We replicate all the main results on this subset of trials.

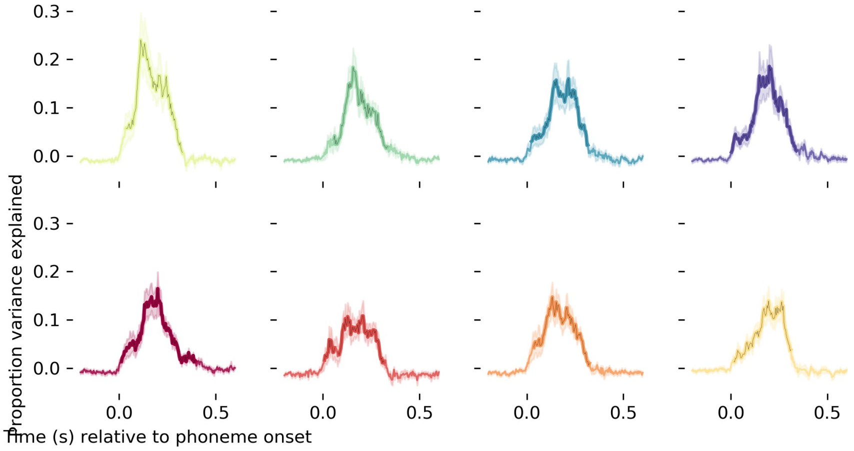

Supplementary Figure 4: **Diagonal decoding on equalised phoneme counts.** Trace represents average decoding performance over 21 participants. Shading represents standard error of the mean over participants.

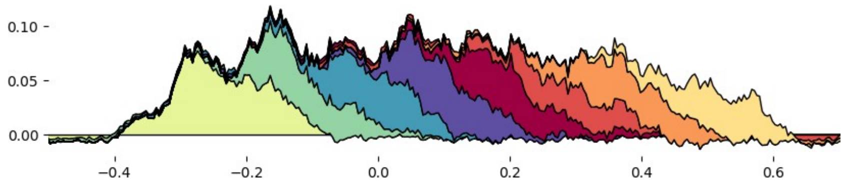

Supplementary Figure 5: **Cumulative diagonal decoding.** Each colour represents one of the 10 phoneme positions. Variance explained is cumulated over phoneme positions.

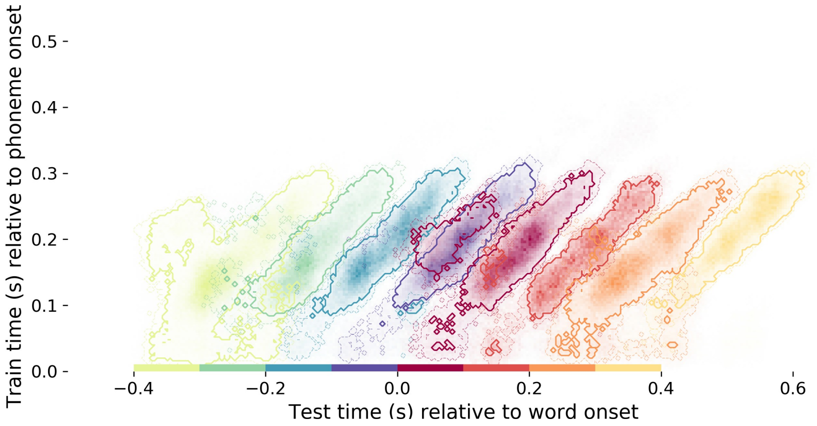

Supplementary Figure 6: **Temporal generalisation decoding on equalised phoneme counts.** Figure details are the same as in Main Figure 3.

Additional analyses of interest

1.3.1. Sequence analysis on mel spectrogram

One of our claims is that there is very little representational overlap between consecutive speech sounds in the neural data, whereas there is a lot of overlap in the auditory signal. Here we show the equivalent of main Figure 3, panels A and B, when applied to the mel spectrogram rather than the MEG responses.

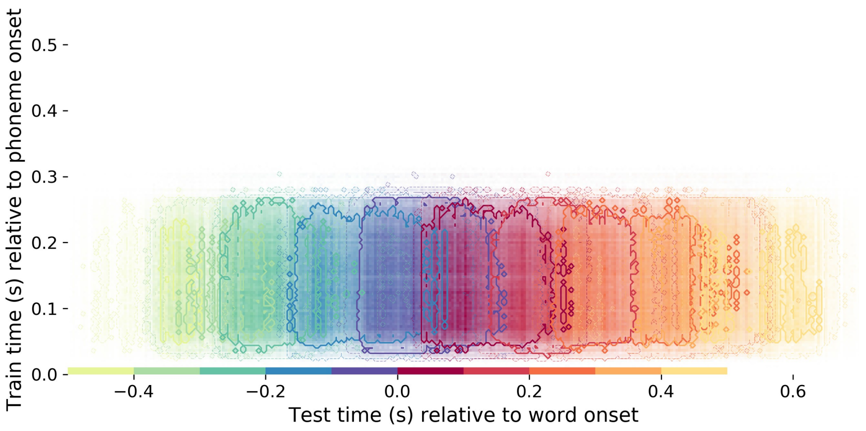

Supplementary Figure 7: **Temporal generalisation analysis of each phoneme position applied to the mel spectrogram.** Figure details are the same as in Main Figure 3.

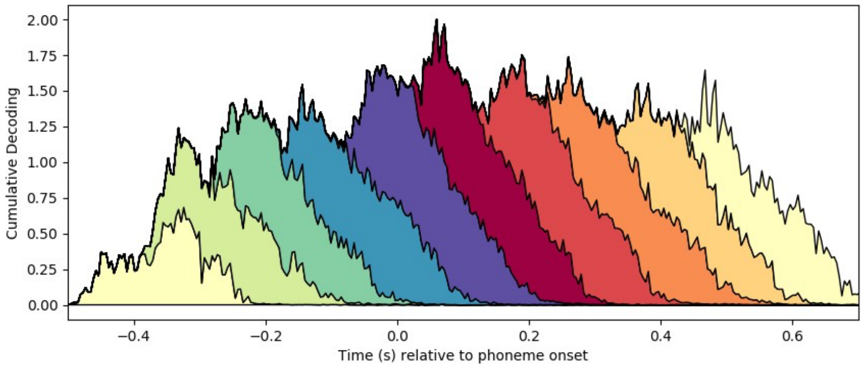

Supplementary Figure 8: **Cumulative decoding performance across the sequence, applied to mel spectrogram.**. Each colour represents a different phoneme position.

1.3.2. History and future decoding

Our results suggest that at the brain processes at least three phonemes concurrently. To add further support to this claim, we decoded the content of three preceding phonemes and three subsequent phonemes from a given moment in test time. As shown in Figure 9, we can indeed decode the phonetic content of three phonemes from the same neural response. This was clearer for voicing and manner than place of articulation, in line with the general trend that place of articulation is not as robustly encoded as the other properties [1, 2]. For the history decoding, decoding performance appears to peak time-locked to the onset of subsequent phonemes, perhaps suggestive of a re-activation procedure. This is in line with previous results from our lab (e.g. [3]).

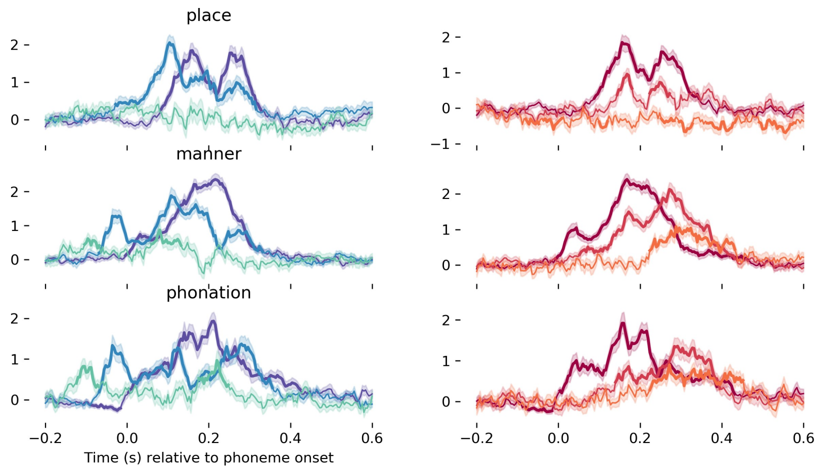

Supplementary Figure 9: **Decoding phonetic history and future from a single neural timecourse.** Trace represents average decoding performance over 21 participants. Shading represents standard error of the mean over participants.

1.3.3. Sequence representation for different phonetic features

A lot of the analyses we applied were aggregated over the individual phonetic features. However, even though the individual feature sequence maps are noisier, they show the same diagonal patterns as the full aggregated data.

There are, however, some interesting dynamics that may be worth exploring in future work. For example, the voicing feature is appears more sustained across positions. And the 'appendage' of the first phoneme appears most pronounced for manner of articulation.

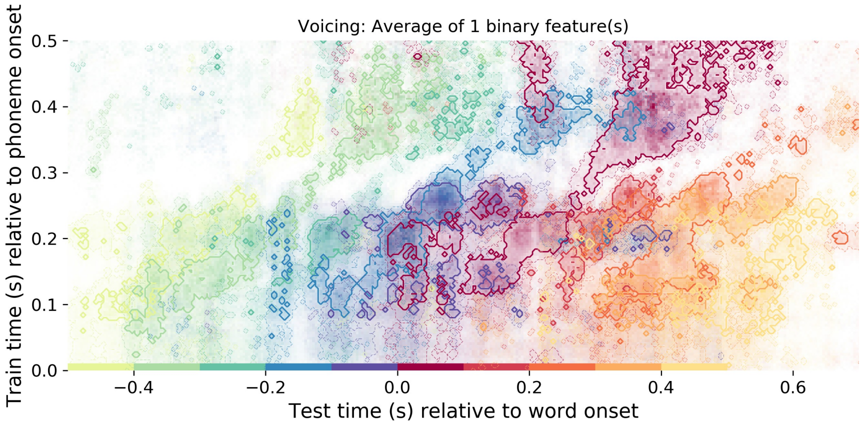

Supplementary Figure 10: **Sequence decoding for voicing.** Figure details are the same as in Main Figure 3.

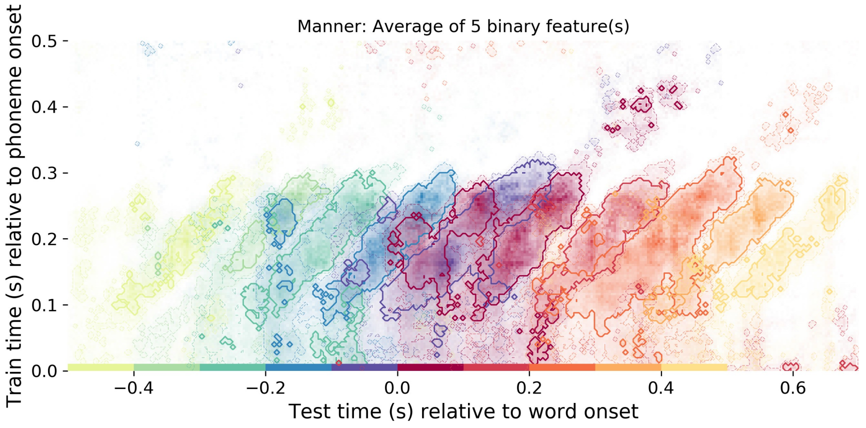

Supplementary Figure 11: **Sequence decoding for manner.** Figure details are the same as in Main Figure 3.

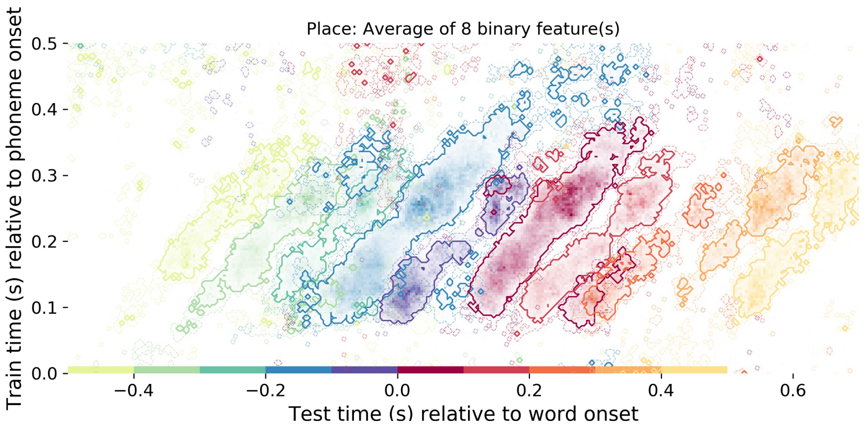

Supplementary Figure 12: **Sequence decoding for place of articulation.** Figure details are the same as in Main Figure 3.

1.3.4. Dynamics at sentence onset and sentence offset

We re-ran the neural generalisation analysis on two subsets of phonemes. First, testing on all phonemes at the onset of words and the onset of sentences (and training on all others). Second, testing on all phonemes at the offset of words and at the offset of sentences (and training on all others). The goal was to test whether (i) the ‘appendage’ observed for the onset phoneme was caused by predictability of the phoneme in context, in which case we should not observe it at the beginning of sentences because there is no context upon which to generate reliable predictions; (ii) whether we still observe ‘diagonal dynamics’ when the phoneme is followed by silence. The model was fit on responses to about 20,000 phonemes and tested on about 500 phonemes. The results show average decoding performance across phonetic features.

Interesting we observe that the ‘appendage’ is still present for onset phonemes at the beginning of sentences. This suggests that the maintenance of phonetic features may be some that always happens at the beginning of words, regardless of how predictable the word is in context. Future work should test the reason for this.

Furthermore we find that the diagonal pattern is also present for phonemes at the end of sentences which are followed by silence. It seems that phonetic detail is actually maintained during the silent period, another fascinating result which should be followed up with subsequent research.

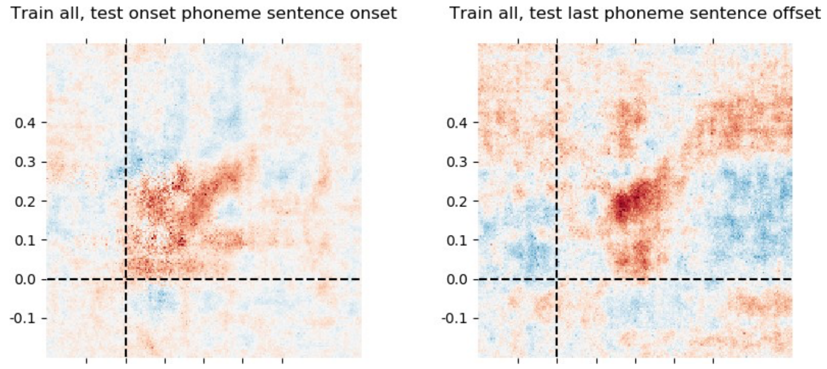

Supplementary Figure 13: **Generalisation analysis applied to the first phoneme of a sentence and the last phoneme of a sentence.** x-axis corresponds to time in seconds relative to training the classifier on all phoneme responses. The y-axis corresponds to time in seconds relative to testing the classifier on responses to the first phoneme in the sentence (left) and the last phoneme in the sentence (right).

1.3.5. Testing granularity of representation

Throughout the manuscript we focus on phonetic features as the way to represent linguistically relevant information about speech sounds. One possibility is that, during the timecourse of processing, representations evolve from more sensory to more abstract - e.g. from acoustics to phonetic features to phoneme categories.

We ran the analysis on the raw (not acoustically residual data), trying to decode (i) spectral features of the phonemes (ii) the same 14 phonetic features that this manuscript is concerned with (iii) one-hot encoding of consonants (e.g. /b/, /p/) (iv) one-hot encoding of vowels (e.g. /ae/, /oo/) and (vi) all the other higher order features that we have analysed in the study. We tested these different formats of representing speech sounds for their relative timecourse of decodability. Unfortunately, the representations are too correlated to make any strong claims in one direction or another, and there was no clear temporal separation between the different formats. Future controlled studies may be able to associate different moments in the processing trajectory with different representational formats, once they have been sufficiently de-correlated in the design

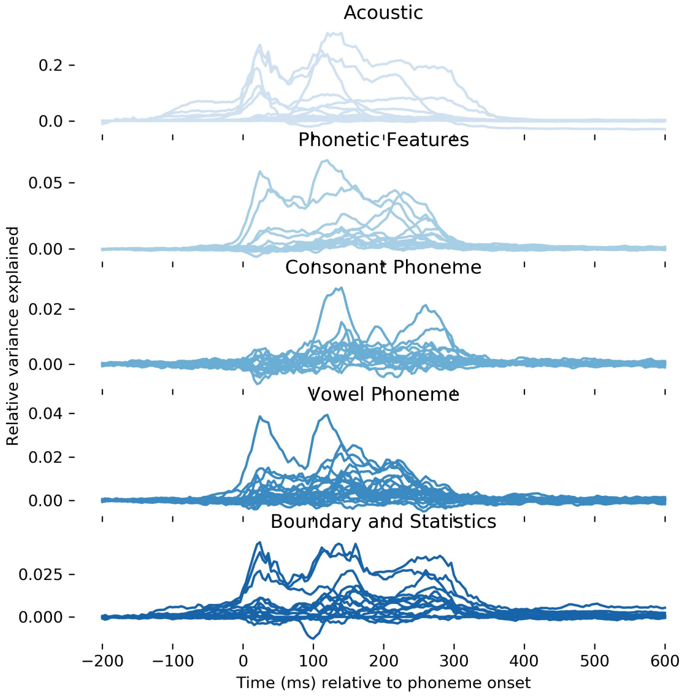

Supplementary Figure 14: **Decoding different speech sound representational formats.** Each trace represents a different feature within the feature family displayed on each row.

Spatial evolution of encoding

1.4.1. Beta coefficients of decoding model

Here we show the coefficients of a linear decoding model averaged across participants, for each of the 31 features. Each participant and each repetition of the story contains 25259 individual phoneme responses.

To evaluate statistical significance we also ran the decoding analysis on shuffled versions of each feature, for each participant and repetition. We computed a metric of trajectory structure which was a weighted combination of range of movement (m, maximum cosine distance minus minimum), smoothness (s, mean absolute step size at each time sample) and variance (v, standard deviation across time samples), thus:

$$\frac{m}{s} v \tag{1}$$

We compute this metric for the x co-ordinates and y co-ordinates separately, and then average the result. To test statistical reliability we ran an independent t-test between the coefficients from the true features and the coefficients from the shuffled features.

Some notable results are the robust position-based trajectories such as phoneme location in the word (True Mean = 1.72, SD = 0.85, Null Mean = 1.06, SD = 0.5, t = 4.25, p < .001) and phonetic feature trajectories such as voicing (True Mean = 1.42, SD = 0.59, Null Mean = 1.04, SD = 0.44, t = 3.24, p < .001), nasality (True Mean = 1.34, SD = 0.57, Null Mean = 1.07, SD = 0.4, t = 2.56, p= 0.01) and fricative (True Mean = 1.32, SD = 0.55, Null Mean = 1.09, SD = 0.38, t = 2.26, p = 0.03).

Our results for all of the 31 features are as follows: Word length: True Mean = 1.09, SD = 0.63, Null Mean = 1.19, SD = 0.5, t = -0.78, p = 0.44. Root Onset: True Mean = 1.7, SD = 0.62, Null Mean = 1.18, SD = 0.49, t = 4.27, p = 0.0. Phon. offset distance: True Mean = 1.28, SD = 0.73, Null Mean = 1.04, SD = 0.44, t = 1.86, p = 0.07. Entropy: True Mean = 1.58, SD = 0.62, Null Mean = 1.05, SD = 0.45, t = 4.43, p = 0.0. Phon. Loc Sentence: True Mean = 0.86, SD = 0.97, Null Mean = 1.11, SD = 0.42, t = -1.51, p = 0.13. Phon. Loc Syllable: True Mean = 1.39, SD = 0.56, Null Mean = 1.03, SD = 0.36, t = 3.44, p = 0.0. Phon. Loc Word: True Mean = 1.72, SD = 0.85, Null Mean = 1.06, SD = 0.5, t = 4.25, p = 0.0. Prefix Onset: True Mean = 1.21, SD = 0.54, Null Mean = 1.18, SD = 0.47, t = 0.34, p = 0.74. Primary Stress: True Mean = 1.52, SD = 0.49, Null Mean = 1.13, SD = 0.5, t = 3.48, p = 0.0. Secondary Stress: True Mean = 1.1, SD = 0.5, Null Mean = 0.95, SD = 0.37, t = 1.56, p = 0.12. Sequence Frequency: True Mean = 1.79, SD = 0.83, Null Mean = 1.09, SD = 0.39, t = 4.85, p = 0.0. Suffix Onset: True Mean = 1.38, SD = 0.51, Null Mean = 0.98, SD = 0.42, t = 3.85, p = 0.0. Surprisal: True Mean = 1.71, SD = 0.71, Null Mean = 0.97, SD = 0.32, t = 6.06, p = 0.0. Syll. Loc Word: True Mean = 1.77, SD = 0.92, Null Mean = 1.09, SD = 0.47, t = 4.23, p = 0.0. Syllable Onset: True Mean = 1.52, SD = 0.43, Null Mean = 1.1, SD = 0.34, t = 4.86, p = 0.0. Word Offset: True Mean = 1.44, SD = 0.62, Null Mean = 1.24, SD = 0.59, t = 1.53, p = 0.13. Word Onset: True Mean = 1.68, SD = 0.57, Null Mean = 1.12, SD = 0.49, t = 4.83, p = 0.0. Approximant: True Mean = 1.27, SD = 0.49, Null Mean = 1.14, SD = 0.38, t = 1.36, p = 0.18. Fricative: True Mean = 1.32, SD = 0.55, Null Mean = 1.09, SD = 0.38, t = 2.26, p = 0.03. Nasal: True Mean = 1.34, SD = 0.57, Null Mean = 1.07, SD = 0.4, t = 2.56, p = 0.01. Plosive: True Mean = 1.33, SD = 0.39, Null Mean = 1.16, SD = 0.44, t = 1.87, p = 0.06. Vowel: True Mean = 1.4, SD = 0.36, Null Mean = 1.11, SD = 0.41, t = 3.38, p = 0.0. Voicing: True Mean = 1.42, SD = 0.59, Null Mean = 1.04, SD = 0.44, t = 3.24, p = 0.0. Glottal: True Mean = 1.06, SD = 0.41, Null Mean = 1.12, SD = 0.5, t = -0.61,

$p = 0.54$. Coronal: True Mean = 1.36, SD = 0.43, Null Mean = 1.15, SD = 0.48, $t = 2.13$, $p = 0.04$. Dental: True Mean = 1.25, SD = 0.49, Null Mean = 1.12, SD = 0.42, $t = 1.34$, $p = 0.18$. High Vowel: True Mean = 1.31, SD = 0.52, Null Mean = 1.06, SD = 0.41, $t = 2.42$, $p = 0.02$. Labial: True Mean = 1.25, SD = 0.51, Null Mean = 1.14, SD = 0.49, $t = 1.01$, $p = 0.31$. Low Vowel: True Mean = 1.18, SD = 0.53, Null Mean = 1.06, SD = 0.41, $t = 1.18$, $p = 0.24$. Middle Vowel: True Mean = 1.19, SD = 0.42, Null Mean = 1.16, SD = 0.54, $t = 0.32$, $p = 0.75$. Velar: True Mean = 1.13, SD = 0.41, Null Mean = 1.09, SD = 0.5, $t = 0.45$, $p = 0.65$.

1.14.1. Trajectory for each feature

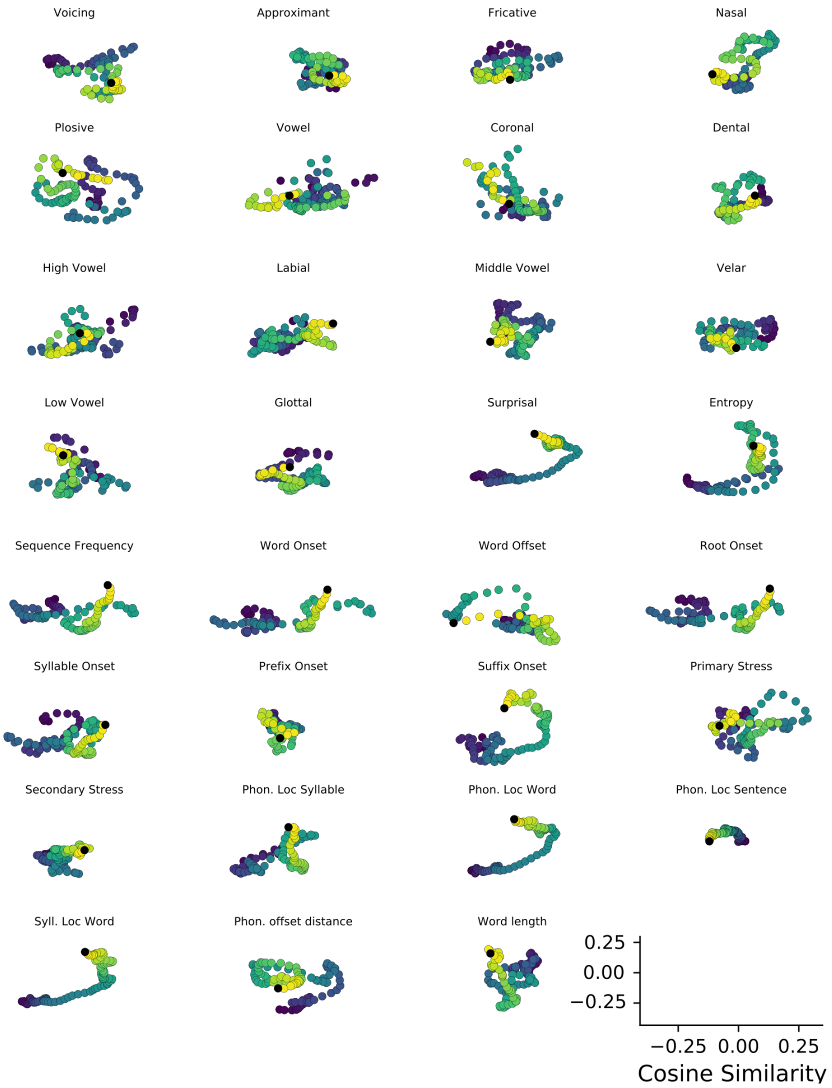

Supplementary Figure 15: **Trajectory for each feature.** Showing the movement of the decoding coefficients within sensor space. Black dot shows the ending point of the trajectory. More purple colours represent earlier moments in time. Details are the same as Main Figure 4B.

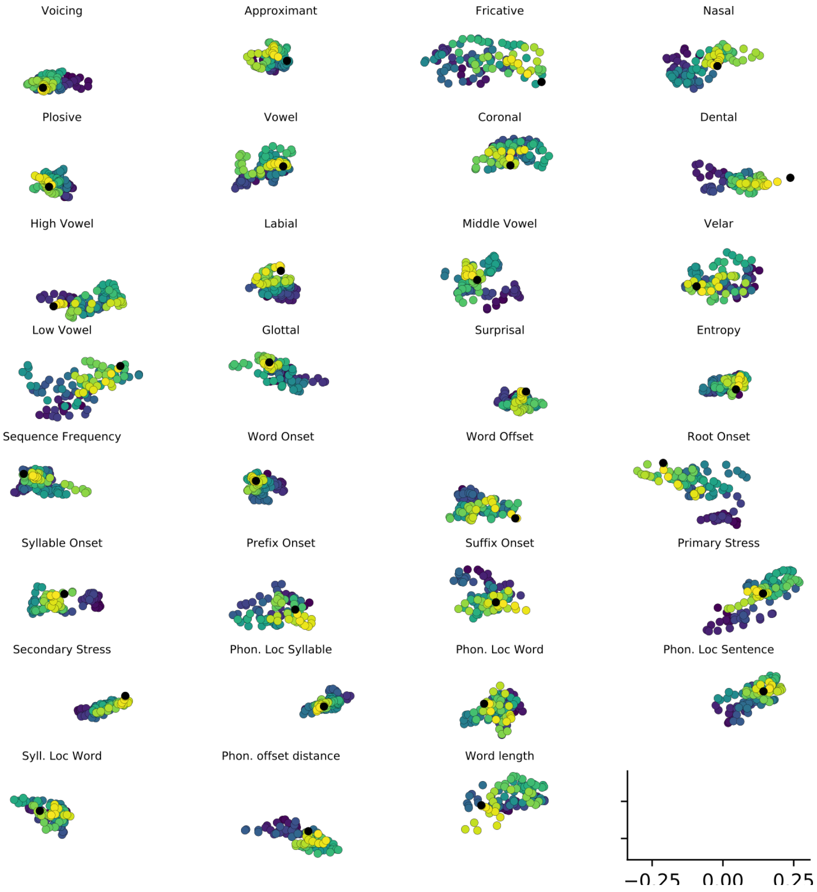

Supplementary Figure 16: **Trajectory when shuffing trial correspondence in decoding (i.e. null trajectory for comparison).** Black dot shows the ending point of the trajectory. More purple colours represent earlier moments in time.

176 *1.14.2. Voicing*

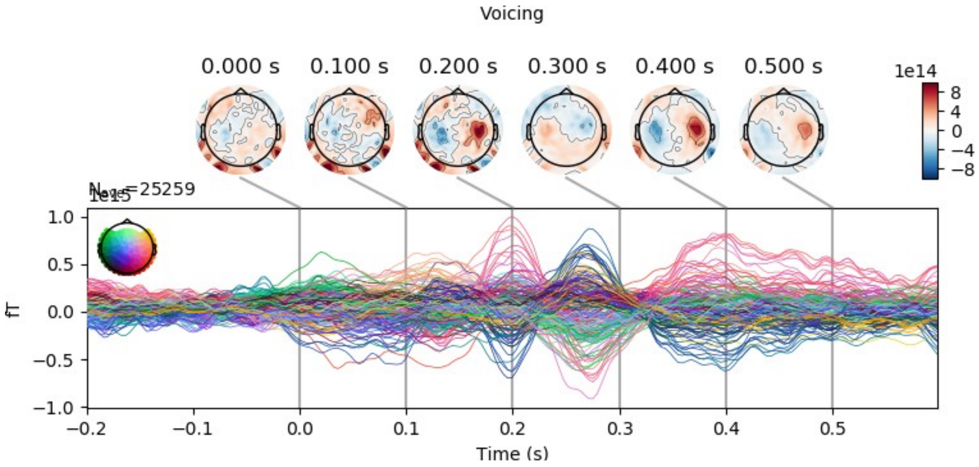

Supplementary Figure 17: **Coefficients for Voicing.** x-axis corresponds to time in seconds relative to phoneme onset. y-axis corresponds to the signed magnitude of the decoding coefficient which maps from sensor space to feature space. Each trace corresponds to a different MEG sensor.

1.14.3. Manner

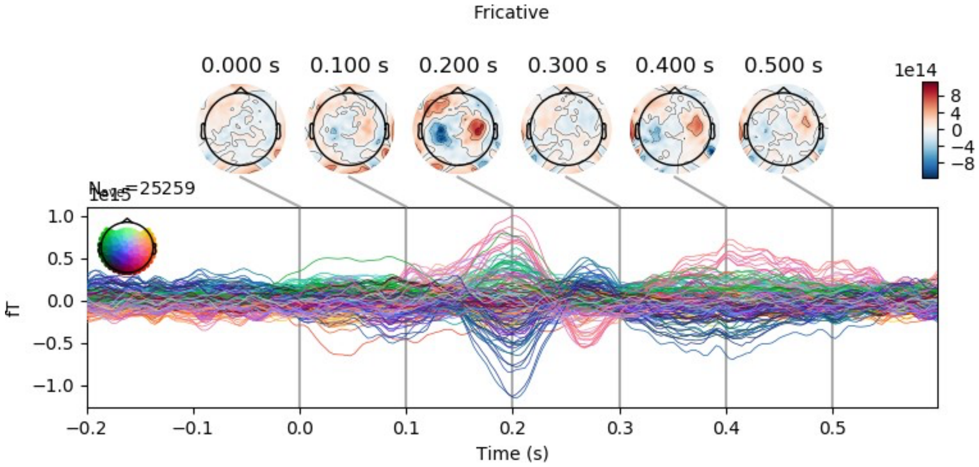

Supplementary Figure 18: **Coefficients for Frication.** x-axis corresponds to time in seconds relative to phoneme onset. y-axis corresponds to the signed magnitude of the decoding coefficient which maps from sensor space to feature space. Each trace corresponds to a different MEG sensor.

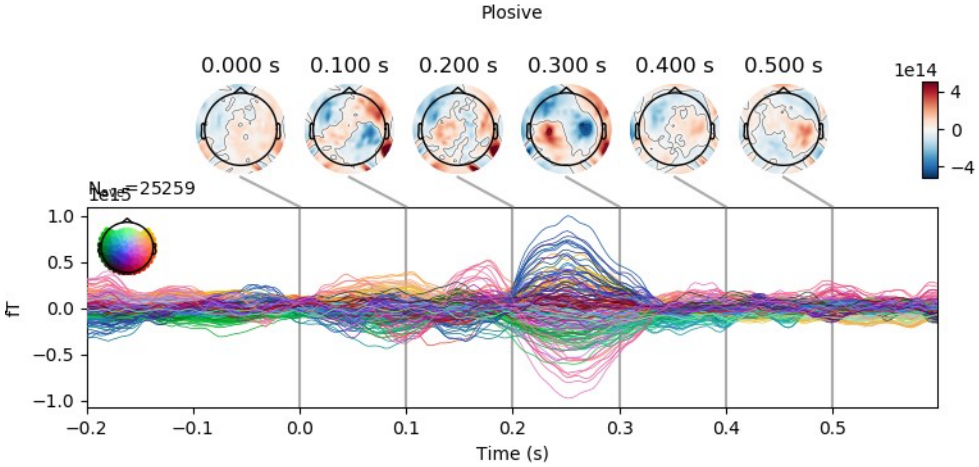

Supplementary Figure 19: **Coefficients for Plosive.** x-axis corresponds to time in seconds relative to phoneme onset. y-axis corresponds to the signed magnitude of the decoding coefficient which maps from sensor space to feature space. Each trace corresponds to a different MEG sensor.

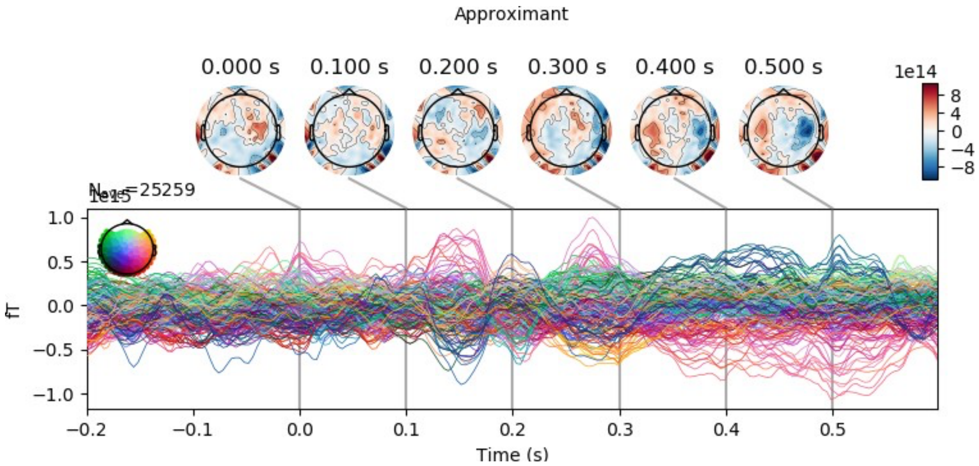

Supplementary Figure 20: **Coefficients for Approximant.** x-axis corresponds to time in seconds relative to phoneme onset. y-axis corresponds to the signed magnitude of the decoding coefficient which maps from sensor space to feature space. Each trace corresponds to a different MEG sensor.

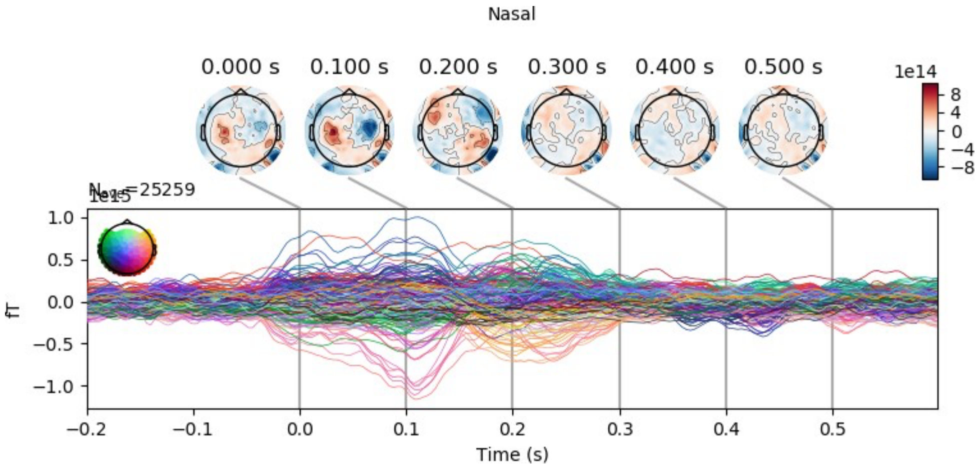

Supplementary Figure 21: **Coefficients for Nasal.** x-axis corresponds to time in seconds relative to phoneme onset. y-axis corresponds to the signed magnitude of the decoding coefficient which maps from sensor space to feature space. Each trace corresponds to a different MEG sensor.

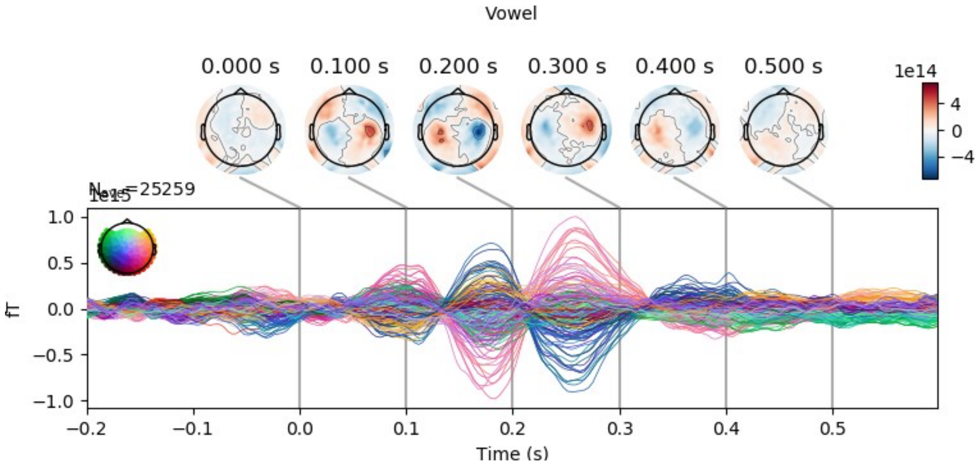

Supplementary Figure 22: **Coefficients for Vowel.** x-axis corresponds to time in seconds relative to phoneme onset. y-axis corresponds to the signed magnitude of the decoding coefficient which maps from sensor space to feature space. Each trace corresponds to a different MEG sensor.

1.14.4. Place of articulation: Consonants

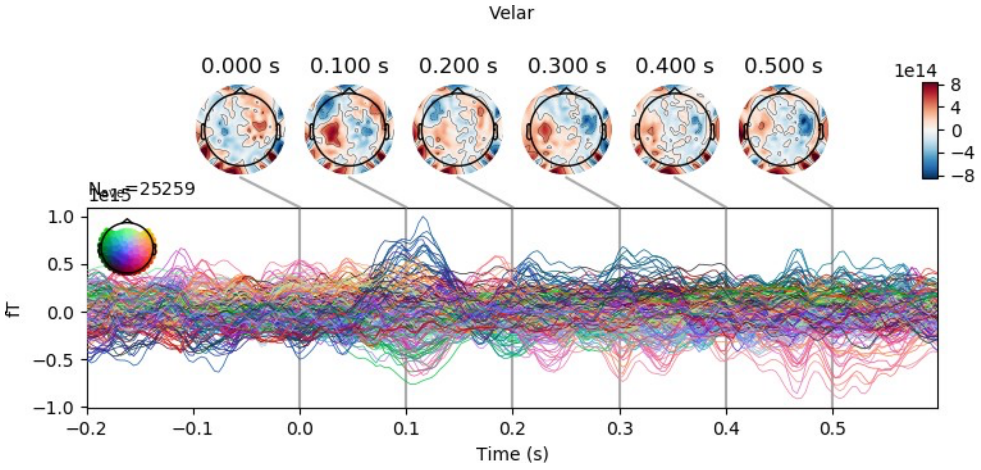

Supplementary Figure 23: **Coefficients for Velar.** x-axis corresponds to time in seconds relative to phoneme onset. y-axis corresponds to the signed magnitude of the decoding coefficient which maps from sensor space to feature space. Each trace corresponds to a different MEG sensor.

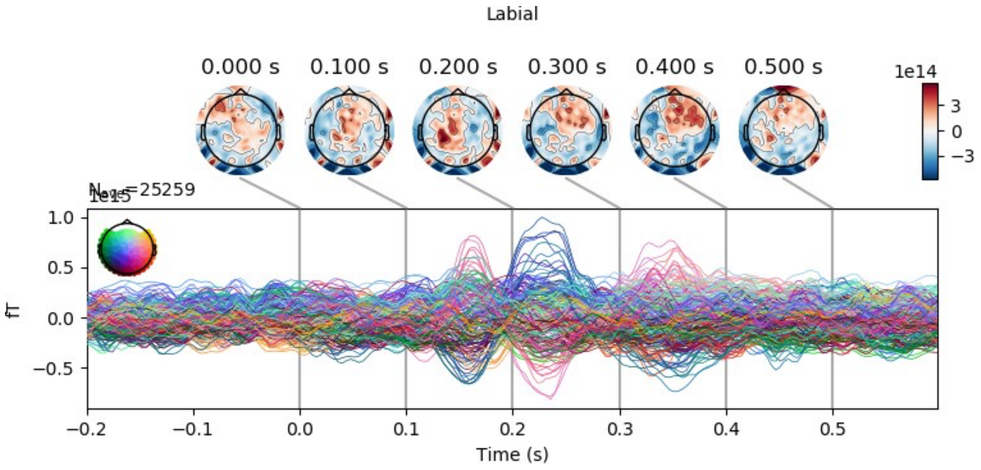

Supplementary Figure 24: **Coefficients for Labial.** x-axis corresponds to time in seconds relative to phoneme onset. y-axis corresponds to the signed magnitude of the decoding coefficient which maps from sensor space to feature space. Each trace corresponds to a different MEG sensor.

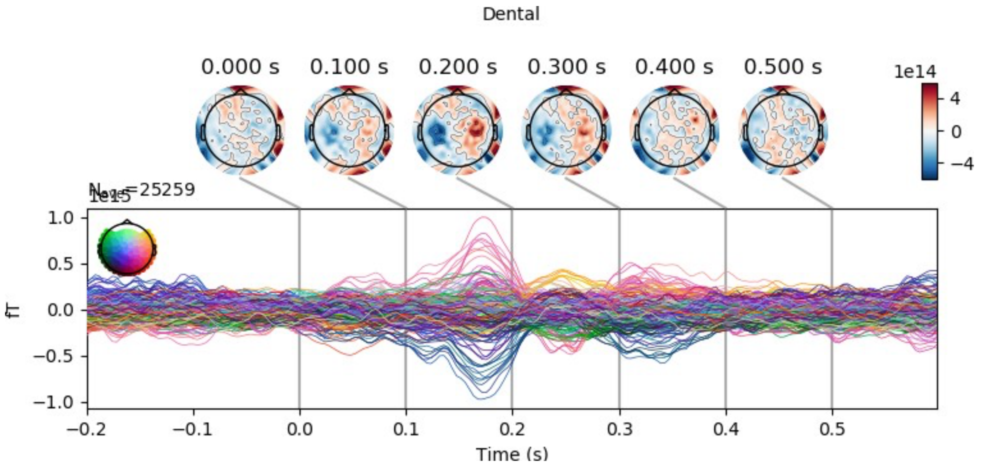

Supplementary Figure 25: **Coefficients for Dental.** x-axis corresponds to time in seconds relative to phoneme onset. y-axis corresponds to the signed magnitude of the decoding coefficient which maps from sensor space to feature space. Each trace corresponds to a different MEG sensor.

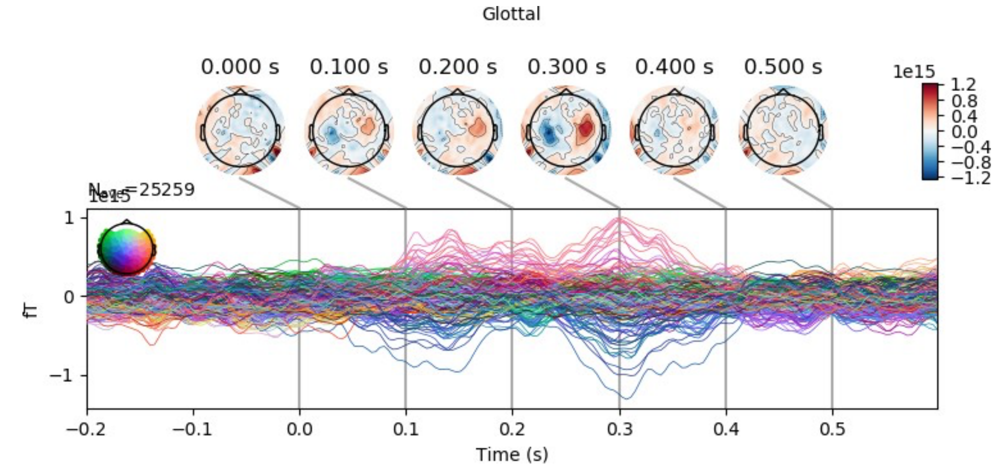

Supplementary Figure 26: **Coefficients for Glottal.** x-axis corresponds to time in seconds relative to phoneme onset. y-axis corresponds to the signed magnitude of the decoding coefficient which maps from sensor space to feature space. Each trace corresponds to a different MEG sensor.

1.14.5. *Place of articulation: Vowels*

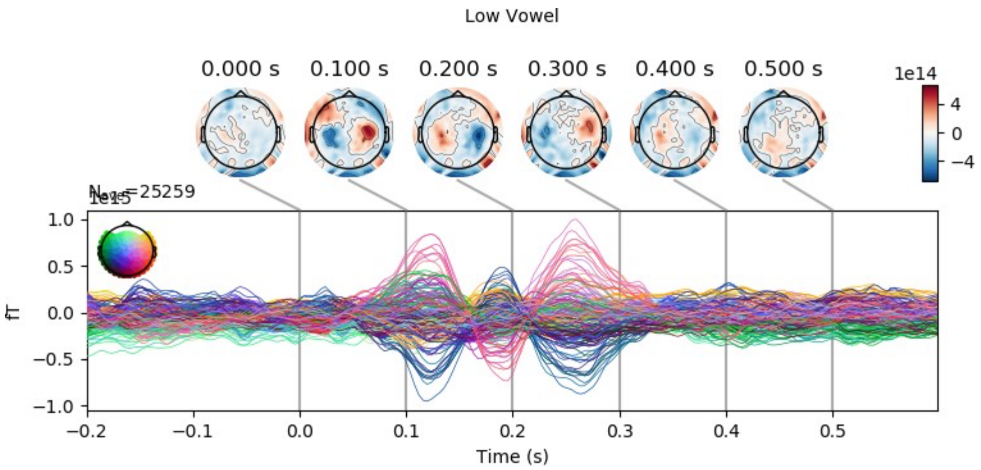

Supplementary Figure 27: **Coefficients for Low Vowel.** x-axis corresponds to time in seconds relative to phoneme onset. y-axis corresponds to the signed magnitude of the decoding coefficient which maps from sensor space to feature space. Each trace corresponds to a different MEG sensor.

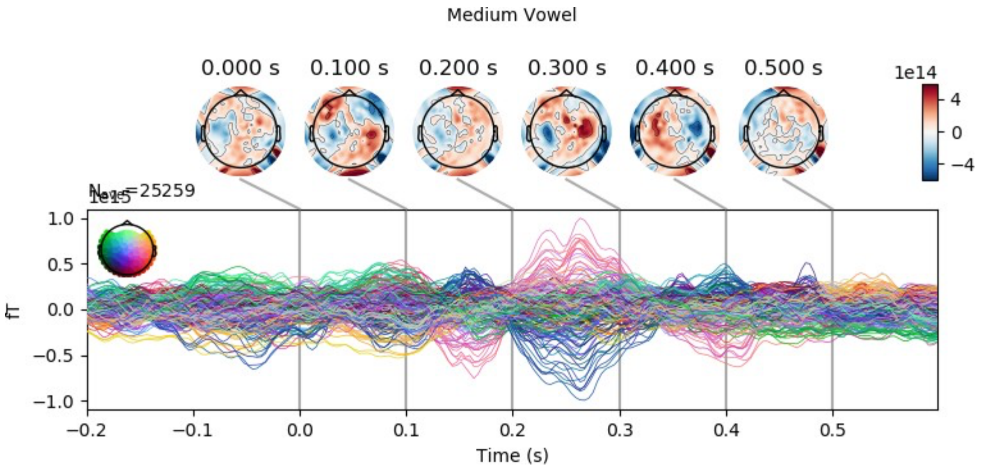

Supplementary Figure 28: **Coefficients for Mid Vowel.** x-axis corresponds to time in seconds relative to phoneme onset. y-axis corresponds to the signed magnitude of the decoding coefficient which maps from sensor space to feature space. Each trace corresponds to a different MEG sensor.

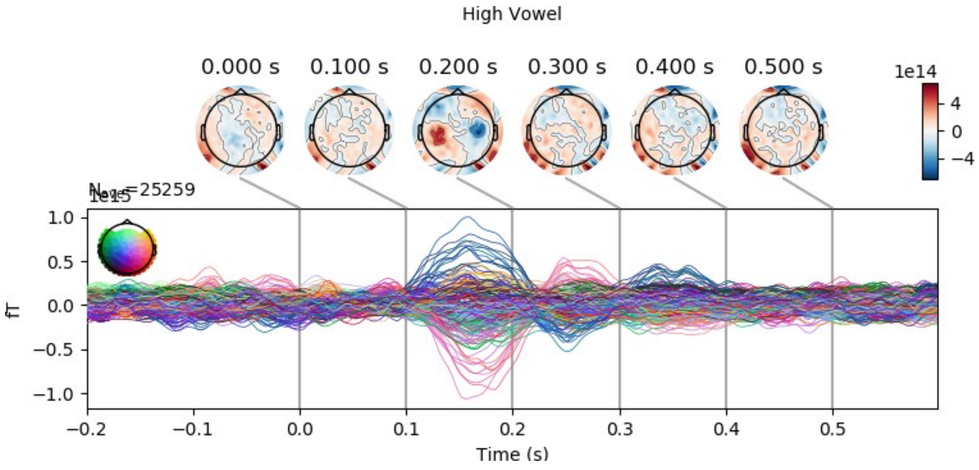

Supplementary Figure 29: **Coefficients for High Vowel.** x-axis corresponds to time in seconds relative to phoneme onset. y-axis corresponds to the signed magnitude of the decoding coefficient which maps from sensor space to feature space. Each trace corresponds to a different MEG sensor.

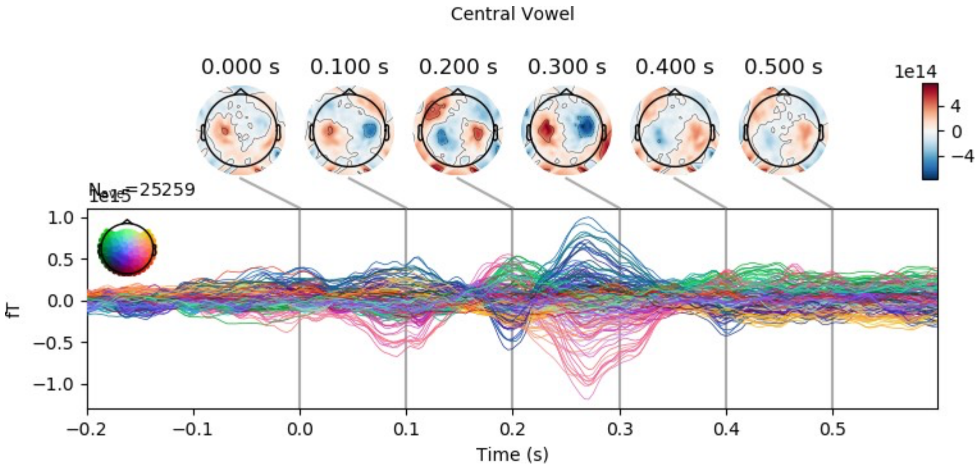

Supplementary Figure 30: **Coefficients for Central Vowel.** x-axis corresponds to time in seconds relative to phoneme onset. y-axis corresponds to the signed magnitude of the decoding coefficient which maps from sensor space to feature space. Each trace corresponds to a different MEG sensor.

1.14.6. Boundary Features

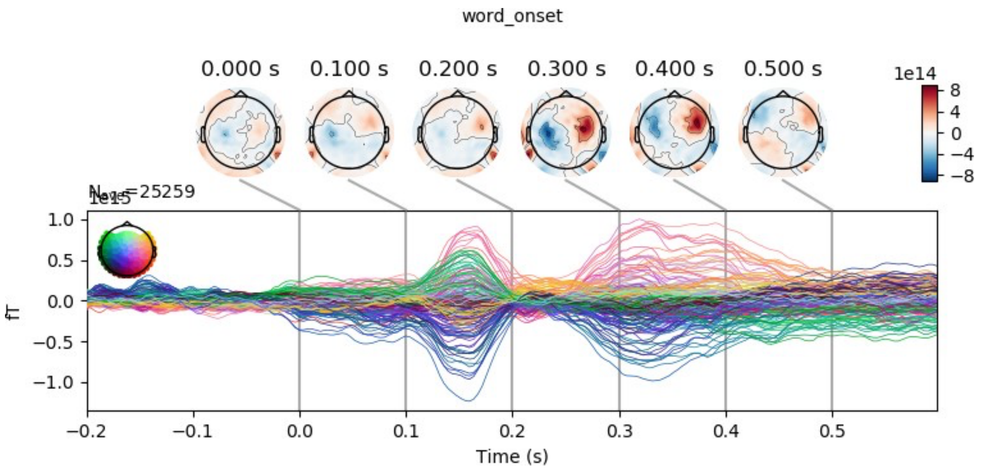

Supplementary Figure 31: **Coefficients for Word Onset.** x-axis corresponds to time in seconds relative to phoneme onset. y-axis corresponds to the signed magnitude of the decoding coefficient which maps from sensor space to feature space. Each trace corresponds to a different MEG sensor.

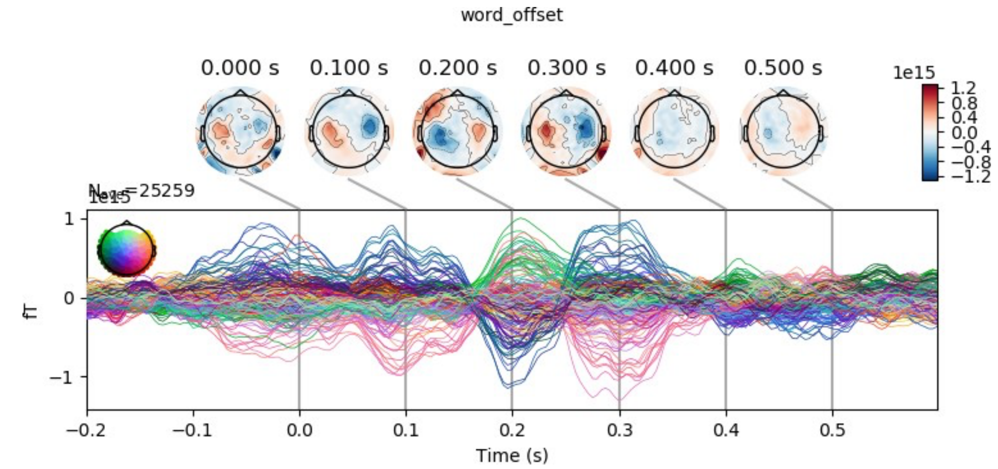

Supplementary Figure 32: **Coefficients for Word O↵set.** x-axis corresponds to time in seconds relative to phoneme onset. y-axis corresponds to the signed magnitude of the decoding coefficient which maps from sensor space to feature space. Each trace corresponds to a different MEG sensor.

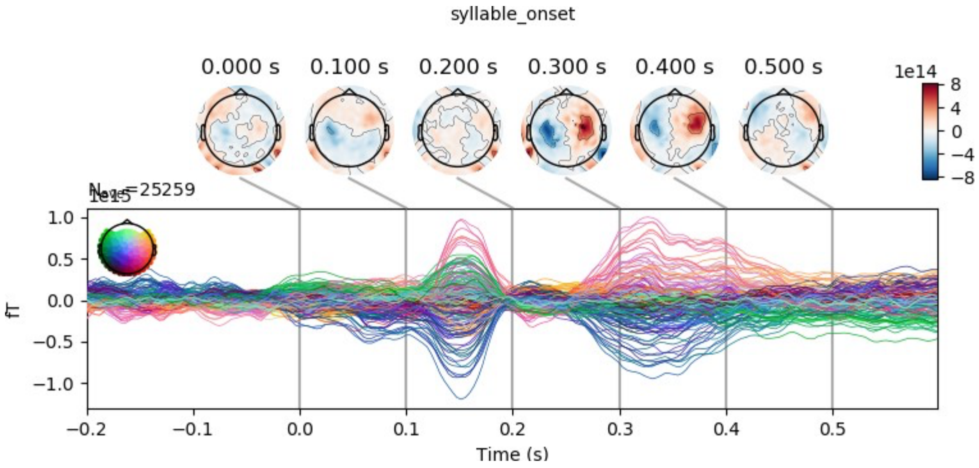

Supplementary Figure 33: **Coefficients for Syllable Onset.** x-axis corresponds to time in seconds relative to phoneme onset. y-axis corresponds to the signed magnitude of the decoding coefficient which maps from sensor space to feature space. Each trace corresponds to a different MEG sensor.

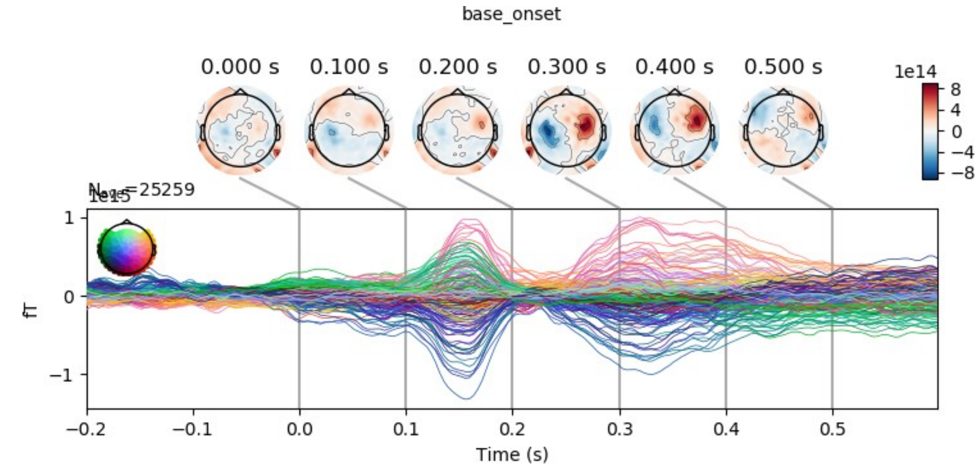

Supplementary Figure 34: **Coefficients for Root Morpheme onset.** x-axis corresponds to time in seconds relative to phoneme onset. y-axis corresponds to the signed magnitude of the decoding coefficient which maps from sensor space to feature space. Each trace corresponds to a different MEG sensor.

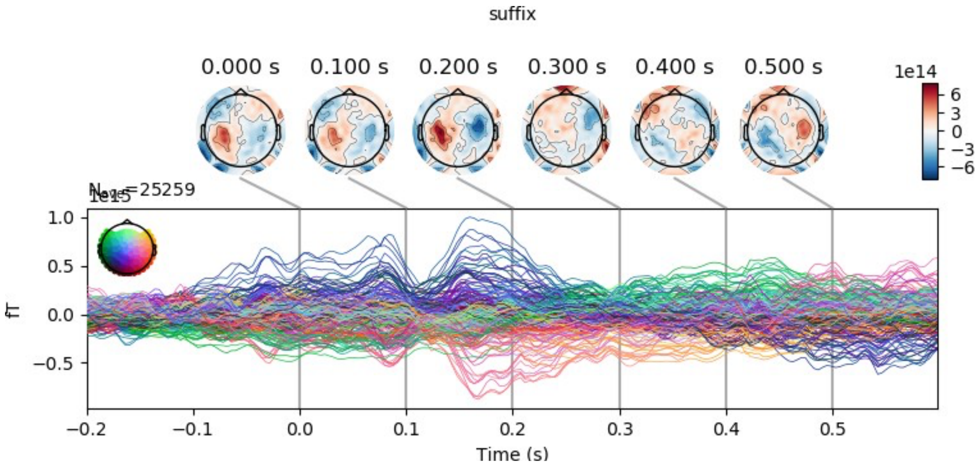

Supplementary Figure 35: **Coefficients for Suffix onset.** x-axis corresponds to time in seconds relative to phoneme onset. y-axis corresponds to the signed magnitude of the decoding coefficient which maps from sensor space to feature space. Each trace corresponds to a different MEG sensor.

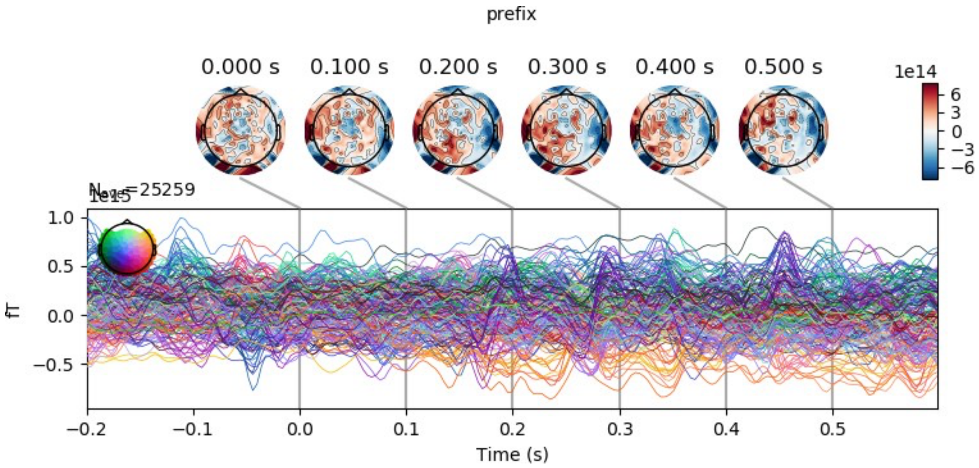

Supplementary Figure 36: **Coefficients for Prefix onset.** x-axis corresponds to time in seconds relative to phoneme onset. y-axis corresponds to the signed magnitude of the decoding coefficient which maps from sensor space to feature space. Each trace corresponds to a different MEG sensor.

1.14.7. Position Features

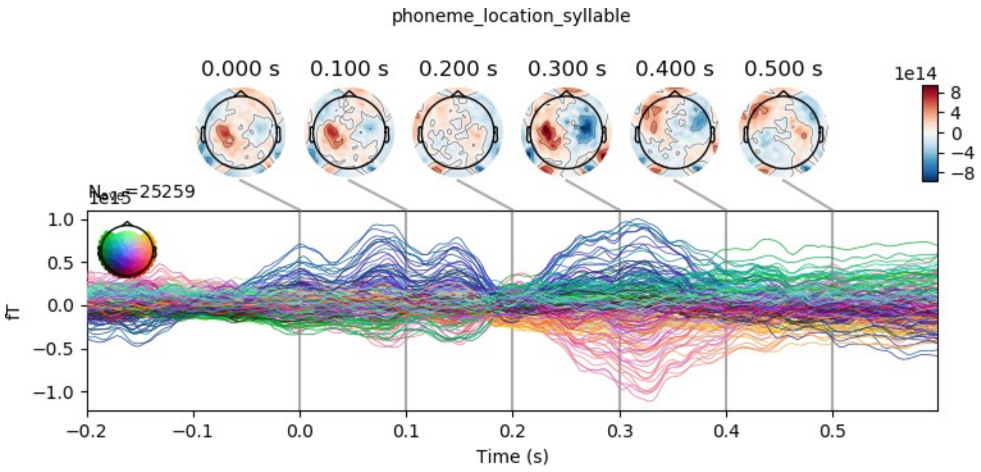

Supplementary Figure 37: **Coefficients for Phoneme location in syllable.** x-axis corresponds to time in seconds relative to phoneme onset. y-axis corresponds to the signed magnitude of the decoding coefficient which maps from sensor space to feature space. Each trace corresponds to a different MEG sensor.

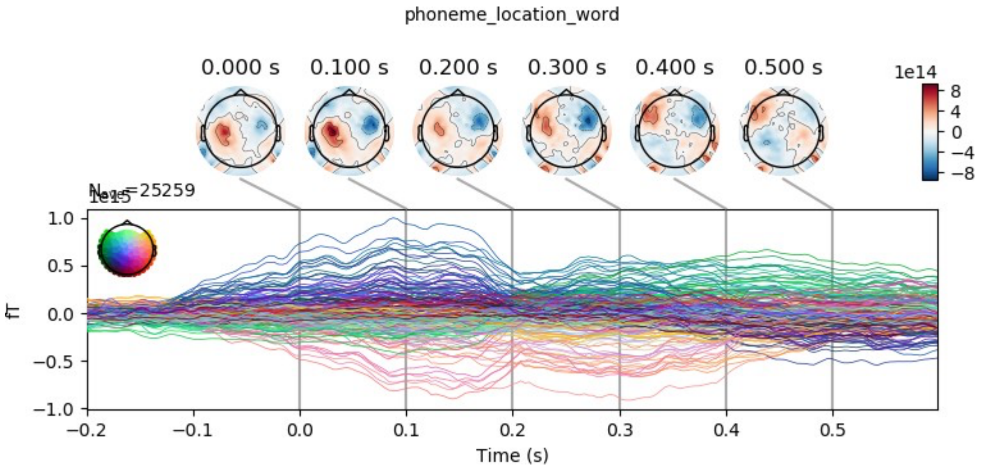

Supplementary Figure 38: **Coefficients for Phoneme location in word.** x-axis corresponds to time in seconds relative to phoneme onset. y-axis corresponds to the signed magnitude of the decoding coefficient which maps from sensor space to feature space. Each trace corresponds to a different MEG sensor.

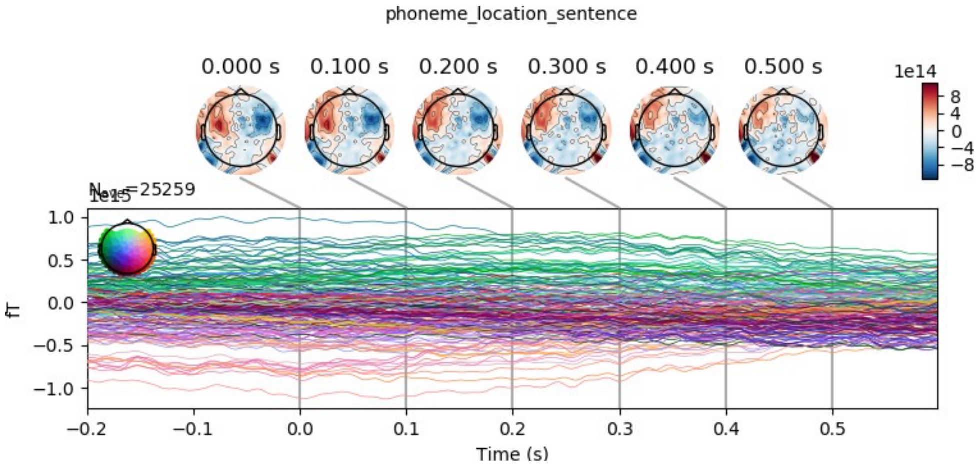

Supplementary Figure 39: **Coefficients for Phoneme location in sentence.** x-axis corresponds to time in seconds relative to phoneme onset. y-axis corresponds to the signed magnitude of the decoding coefficient which maps from sensor space to feature space. Each trace corresponds to a different MEG sensor.

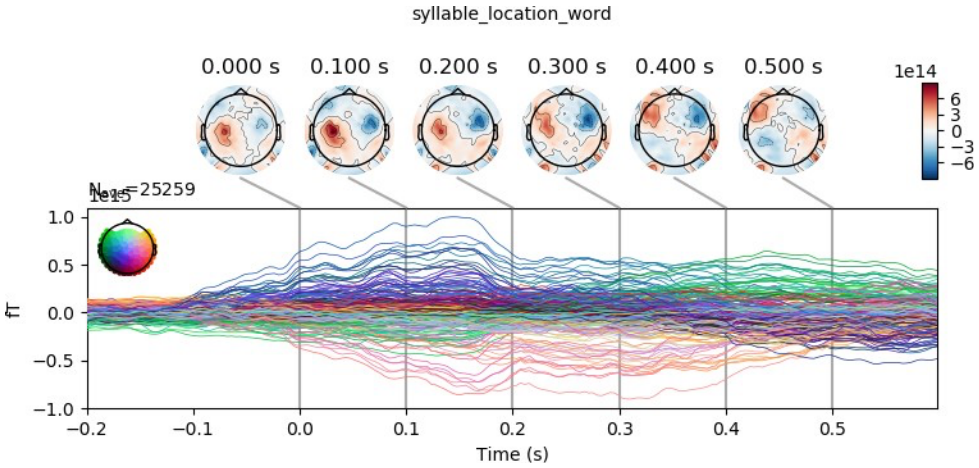

Supplementary Figure 40: **Coefficients for Syllable location in word.** x-axis corresponds to time in seconds relative to phoneme onset. y-axis corresponds to the signed magnitude of the decoding coefficient which maps from sensor space to feature space. Each trace corresponds to a different MEG sensor.

1.14.8. Lexical stress

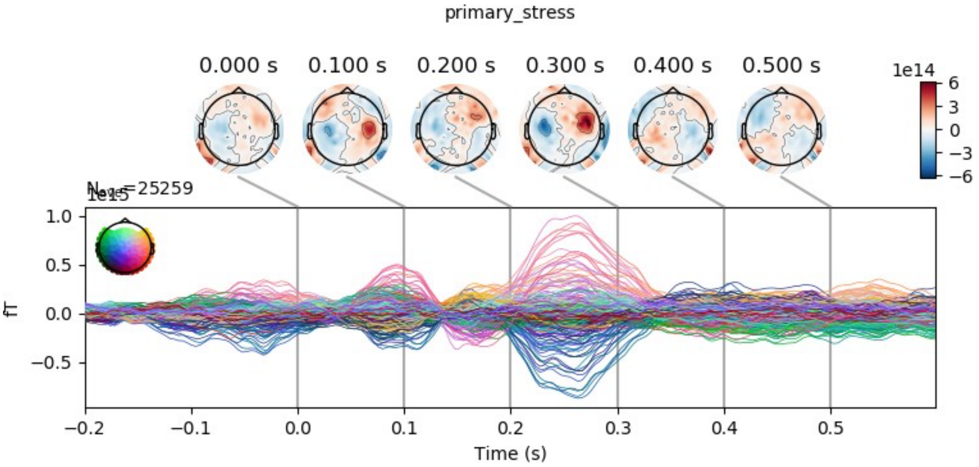

Supplementary Figure 41: **Coefficients for Primary Stress on syllable.** x-axis corresponds to time in seconds relative to phoneme onset. y-axis corresponds to the signed magnitude of the decoding coefficient which maps from sensor space to feature space. Each trace corresponds to a different MEG sensor.

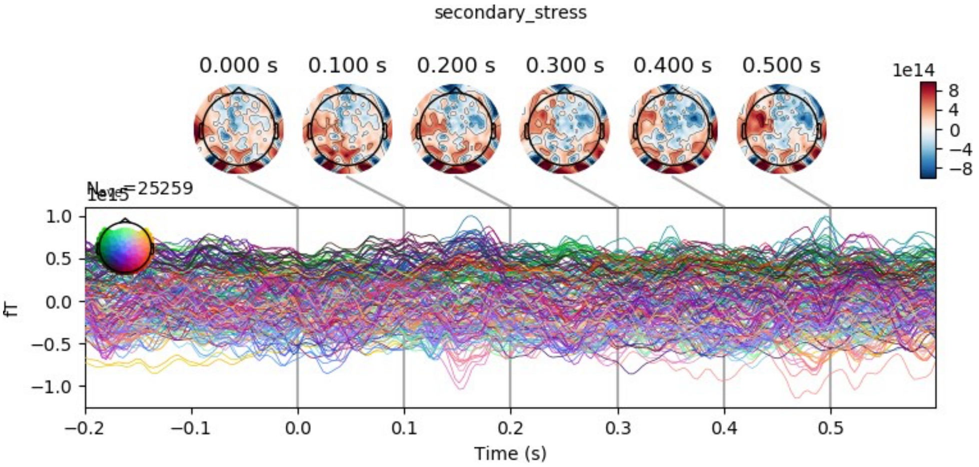

Supplementary Figure 42: **Coefficients for Secondary Stress on syllable.** x-axis corresponds to time in seconds relative to phoneme onset. y-axis corresponds to the signed magnitude of the decoding coefficient which maps from sensor space to feature space. Each trace corresponds to a different MEG sensor.

1.14.9. *Statistical Features*

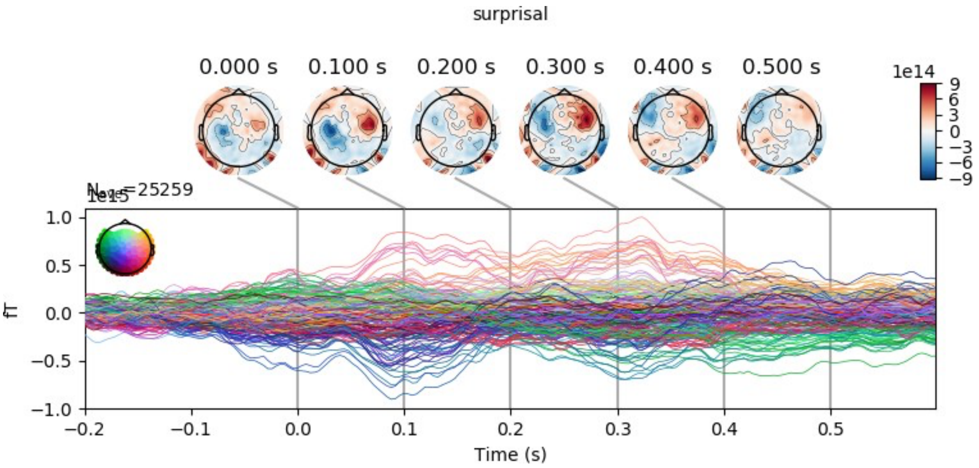

Supplementary Figure 43: **Coefficients for Surprisal.** x-axis corresponds to time in seconds relative to phoneme onset. y-axis corresponds to the signed magnitude of the decoding coefficient which maps from sensor space to feature space. Each trace corresponds to a different MEG sensor.

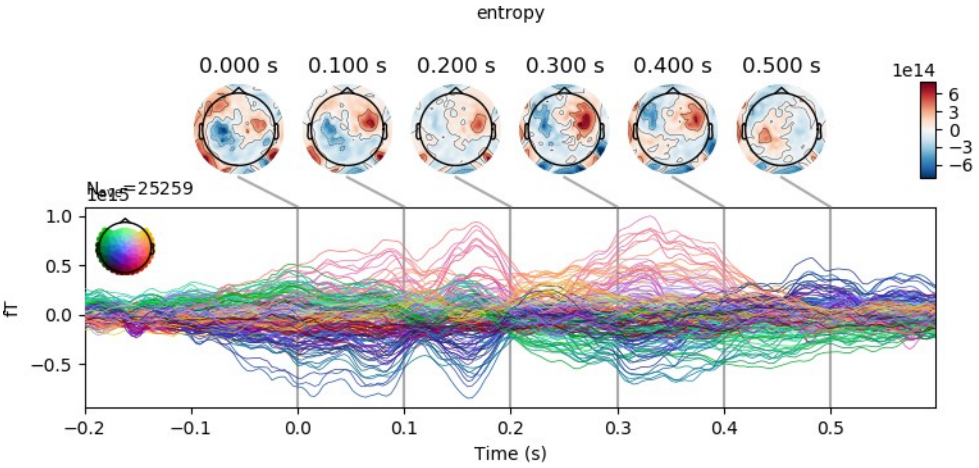

Supplementary Figure 44: **Coefficients for Entropy.** x-axis corresponds to time in seconds relative to phoneme onset. y-axis corresponds to the signed magnitude of the decoding coefficient which maps from sensor space to feature space. Each trace corresponds to a different MEG sensor.

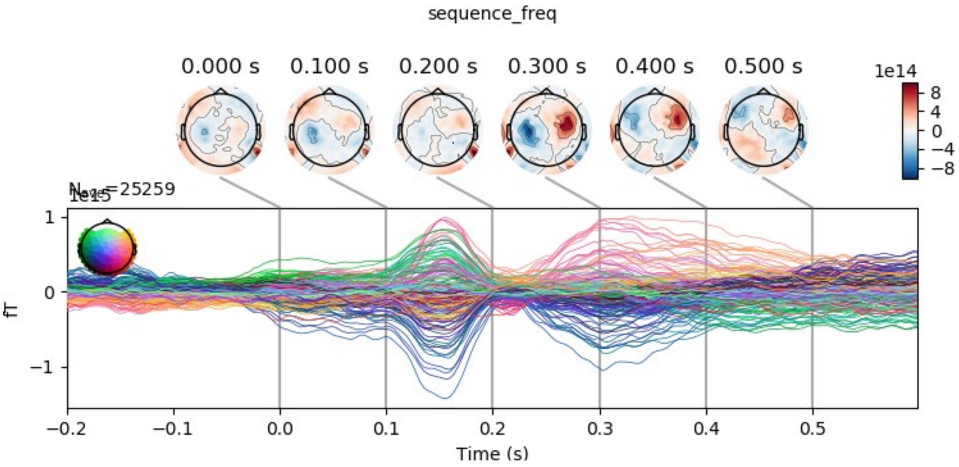

Supplementary Figure 45: **Coefficients for Sequence Frequency.** x-axis corresponds to time in seconds relative to phoneme onset. y-axis corresponds to the signed magnitude of the decoding coefficient which maps from sensor space to feature space. Each trace corresponds to a different MEG sensor.

References

[1] Mesgarani, N., Cheung, C., Johnson, K. & Chang, E. F. Phonetic feature encoding in human superior temporal gyrus. *Science* **343**, 1006–1010 (2014).

[2] Khalighinejad, B., da Silva, G. C. & Mesgarani, N. Dynamic encoding of acoustic features in neural responses to continuous speech. *Journal of Neuroscience* **37**, 2176–2185 (2017).

[3] Gwilliams, L., Linzen, T., Poeppel, D. & Marantz, A. In spoken word recognition, the future predicts the past. *Journal of Neuroscience* **38**, 7585–7599 (2018).
