# Supplementary material for: Neural dynamics of phoneme sequences reveal position-invariant code for content and order
Source: Nat Commun. 2022 Nov 3;13:6606. doi: 10.1038/s41467-022-34326-1 (PMC9633780; doi:10.1038/s41467-022-34326-1)
Supplement: Supplementary file 3 — Reporting Summary [file 41467_2022_34326_MOESM3_ESM.pdf]

nature portfolio

Corresponding author(s): Laura Gwilliams

Last updated by author(s): Sep 5, 2022

Reporting Summary

Nature Portfolio wishes to improve the reproducibility of the work that we publish. This form provides structure for consistency and transparency in reporting. For further information on Nature Portfolio policies, see our Editorial Policies and the Editorial Policy Checklist.

Statistics

For all statistical analyses, confirm that the following items are present in the figure legend, table legend, main text, or Methods section.

n/a	Confirmed	
☐	☒	The exact sample size (*n*) for each experimental group/condition, given as a discrete number and unit of measurement
☐	☒	A statement on whether measurements were taken from distinct samples or whether the same sample was measured repeatedly
☐	☒	The statistical test(s) used AND whether they are one- or two-sided<br>*Only common tests should be described solely by name; describe more complex techniques in the Methods section.*
☐	☒	A description of all covariates tested
☐	☒	A description of any assumptions or corrections, such as tests of normality and adjustment for multiple comparisons
☐	☒	A full description of the statistical parameters including central tendency (e.g. means) or other basic estimates (e.g. regression coefficient) AND variation (e.g. standard deviation) or associated estimates of uncertainty (e.g. confidence intervals)
☐	☒	For null hypothesis testing, the test statistic (e.g. *F*, *t*, *r*) with confidence intervals, effect sizes, degrees of freedom and *P* value noted<br>*Give P values as exact values whenever suitable.*
☒	☐	For Bayesian analysis, information on the choice of priors and Markov chain Monte Carlo settings
☒	☐	For hierarchical and complex designs, identification of the appropriate level for tests and full reporting of outcomes
☐	☒	Estimates of effect sizes (e.g. Cohen's *d*, Pearson's *r*), indicating how they were calculated

Our web collection on statistics for biologists contains articles on many of the points above.

Software and code

Policy information about availability of computer code

Data collection	Data were acquired using the Presentation software (Neuro Behavioural Systems) using version 17.0 and MEG160 (version 1.0; Yokohawa Electric Corporation and Eagle Technology Corporation)
Data analysis	Data were analysed primarily using open source scripts from MNE-Python (version 16.0) and Scikit-learn (version 18.0).

For manuscripts utilizing custom algorithms or software that are central to the research but not yet described in published literature, software must be made available to editors and reviewers. We strongly encourage code deposition in a community repository (e.g. GitHub). See the Nature Portfolio guidelines for submitting code & software for further information.

Data

Policy information about availability of data

All manuscripts must include a data availability statement. This statement should provide the following information, where applicable:

- Accession codes, unique identifiers, or web links for publicly available datasets
- A description of any restrictions on data availability
- For clinical datasets or third party data, please ensure that the statement adheres to our policy

We have uploaded the raw data in BIDS formant to the Open Science Framework (OSF): https://doi.org/10.17605/OSF.IO/AG3KJ. The first 21 subjects in the database correspond to the data used in this study. The subsequent 6 subjects were collected later in time, in order to create a large database for public use by the community. The stimuli we use were obtained from the Open American National Corpus.

Human research participants

Policy information about studies involving human research participants and Sex and Gender in Research.

Reporting on sex and gender	We collected biological sex of our participants, and not their gender identity. We describe the number of male and female (as assigned at birth) participants in our study. Sex was self-reported. We did not perform any aggregation by sex, and do not expect the low-level auditory processes we study here to be different in males and females.
Population characteristics	Twenty-one native English participants (13 female; age: M=24.8, SD=6.4). All participants reported having normal hearing and no history of neurological disorders.
Recruitment	Subjects were recruited through a university-internal system. Most subjects were undergraduate students or researchers at the university. It is unlikely that self-selection bias would affect our results, given the low-level neural processes we are targetting here.
Ethics oversight	The experiment was approved by the IRB ethics committee at New York University Abu Dhabi.

Note that full information on the approval of the study protocol must also be provided in the manuscript.

Field-specific reporting

Please select the one below that is the best fit for your research. If you are not sure, read the appropriate sections before making your selection.

- [x] Life sciences
- [ ] Behavioural & social sciences
- [ ] Ecological, evolutionary & environmental sciences

For a reference copy of the document with all sections, see nature.com/documents/nr-reporting-summary-flat.pdf

Life sciences study design

All studies must disclose on these points even when the disclosure is negative.

Sample size	21 human subjects were recruited. This sample size was selected based on previous experiments we have conducted using the same MEG machine (e.g. Gwilliams & Marantz, 2015, Brain and Language; Gwilliams, Lewis & Marantz, 2018, NeuroImage).
Data exclusions	No data were excluded.
Replication	Subjects completed two sessions of data recording, on two separate days. We confirm that the results we observe in session 1 are replicated for session 2.
Randomization	Subjects listened to stories in the MEG. The assignment of subject to story-order was crossed balanced with a latin-square design. No other randomisation was used.
Blinding	There were no important subject-group allocations in our study.

Reporting for specific materials, systems and methods

We require information from authors about some types of materials, experimental systems and methods used in many studies. Here, indicate whether each material, system or method listed is relevant to your study. If you are not sure if a list item applies to your research, read the appropriate section before selecting a response.

Materials & experimental systems

n/a	Involved in the study	
[x]	[ ]	Antibodies
[x]	[ ]	Eukaryotic cell lines
[x]	[ ]	Palaeontology and archaeology
[x]	[ ]	Animals and other organisms
[x]	[ ]	Clinical data
[x]	[ ]	Dual use research of concern

Methods

n/a	Involved in the study	
[x]	[ ]	ChIP-seq
[x]	[ ]	Flow cytometry
[x]	[ ]	MRI-based neuroimaging
